# Patient knowledge of surgical informed consent and shared decision-making process among surgical patients in Ethiopia: a systematic review and meta-analysis

**DOI:** 10.1186/s13037-023-00386-5

**Published:** 2024-01-13

**Authors:** Mengistu Mera Mihiretu, Ermias Bekele, Kokeb Ayele, Lakew Asmare, Fekade Demeke Bayou, Mastewal Arefaynie, Yawkal Tsega, Abel Endawkie, Shimels Derso Kebede, Natnael Kebede

**Affiliations:** 1 Department of Health System and Management, School of Public Health, College of Medicine and Health Sciences, Wollo University, Dessie, 1145 Ethiopia; 2 Department of Health Informatics, School of Public Health, College of Medicine and Health Sciences, Wollo University, Dessie, Ethiopia; 3 Department of Health Promotion, School of Public Health, College of Medicine and Health Sciences, Wollo University, Dessie, Ethiopia; 4 Department of Epidemiology and Biostatistics, School of Public Health, College of Medicine and Health Sciences, Wollo University, Dessie, Ethiopia; 5 Department of Reproductive and Family Health, School of Public Health, College of Medicine and Health Sciences, Wollo University, Dessie, Ethiopia

**Keywords:** Knowledge, Perception, Informed consent, Factors, Surgical patients, Ethiopia

## Abstract

**Background:**

Informed consent is one of the safeguarding of the patient in medical practice at different standards such as ethical, legal, and administrative purposes. Patient knowledge and perception of informed consent are one of the priority concerns in surgical procedures. Patient knowledge and perception towards informed consent increased patient satisfaction, feeling high power on their determination, and accountability for the management, and facilitated positive treatment outcomes. Despite this, in Ethiopia, there are small-scale primary studies with inconsistent and inconclusive findings. Therefore, this systematic review and meta-analysis study estimated the pooled prevalence of patient knowledge and perception of informed consent and its determinants in Ethiopia.

**Methods:**

We searched major databases such as PubMed, Hinary, MEDLINE, Cochrane Library, EMBASE, Scopus, African Journal Online (AJO), Semantic Scholar, Google Scholar, google, and reference lists. Besides this, University databases in the country were also searched from August 20, 2023, until September 30, 2023,. All published and unpublished studies that report the prevalence of patient knowledge and perception toward informed consent and its associated factors were included. All studies reported in English were included. Studies conducted between January 01, 2015 to September 30, 2023 were included. There are three outcome measurements pooled level of patient knowledge towards informed consent, pooled level of patient perception towards informed consent, and pooled effect that affects patient knowledge of informed consent. Three reviewers (MMM, NK, and YT) independently screened the articles that fulfilled the inclusion criteria to avoid the risk of bias. The studies’ quality was appraised using a modified Newcastle-Ottawa Scale (NOS) version.

**Results:**

The pooled prevalence of appropriate patient knowledge and perception towards informed consent was 32% (95% CI: 21, 43) and 40% (95% CI: 16, 65) respectively. Having formal education 2.69 (95% CI: 1.18, 6.15) and having a history of signed informed consent before 3.65 (95% CI:1.02,13.11) had a statistically significant association with good patient knowledge towards informed consent.

**Conclusion:**

The appropriate patient knowledge and perception of informed consent in Ethiopia is low. Formal education and history of signed informed consent were positive factors for appropriate patient knowledge of informed consent in Ethiopia. Physicians, policymakers, and health facility managers should focus on patients without prior experience with signed informed consent and not have formal education to improve patient knowledge towards informed consent. The protocol was registered at Prospero with number CRD42023445409 and is available from: https://www.crd.york.ac.uk/PROSPERO/#myprospero.

**Supplementary Information:**

The online version contains supplementary material available at 10.1186/s13037-023-00386-5.

## Introduction

Informed consent among patients undergoing surgical procedure is the process of shared decision-making made by the client or his/her surrogates after fully explained what he/she is consenting [1]. It is a voluntary agreement by a competent individual after adequate information regarding the procedure performed, potential benefits and risks, and alternative options of management to make decisions without corrosion [2]. One of the medical practices associated with high risks that require informed consent is surgical invasive procedures [3]. The patient has the right to obtain appropriate expression of all risks and benefits, type of producer, options of treatment, and consequences with scientific justification and evidence [4]. One of the fundamental pillars of surgical treatment is the patient’s informed consent [5].

A globally recognized safeguard for clients undergoing invasive procedures is informed consent [6]. The requirement to make informed consent is patient autonomy (a) the ability of the client to self-determination regarding the procedure that will be done on his/her body. It is self-rule and choice regarding what treatment options physicians propose [2, 7]. Patient comprehension (b) the ability of the client to understand what is explained by health care providers [7]. Adequate information (c) means health care provider disclose in sufficient detail the diagnosis, prognosis, treatment option, potential risks, and benefits by using understandable language to his/her expert decision [2, 8]. Competency (d) the capacity of the client to understand the information, voluntariness (e) decision of consent based on the information rather than coercion, consent (f) agreement between the patient and treating clinician in the proposed treatment procedure with full understanding. Consent form (g) is a written document signed by the client before the surgical procedure [9–11]. Informed consent is the safeguarding of the patient in medical practice at different standards such as ethical, legal, and administrative purposes [2, 6, 12]. The informed consent document builds trust between patients and physicians and enhances the shared decision-making of the client in the surgical procedure. All surgeons check the informed consent document before entering into operation room. Any invasive procedure without signed consent is illegal as well as unethical [12].

Knowledge and perception of the client towards informed consent in the primary study were assessed in the composite variable. Knowledge of informed consent is measured by the know reason why they had surgery, the option of alternative treatment, type of surgery, anesthesia-related risks, postoperative care, the complication of surgery, the legal requirement of informed consent, the right to change their mind after sign, and who protects [13, 14]. Different literature indicated that patient knowledge of informed consent is low. Research conducted in Benin indicated that one-third of the clients (32.3%) experienced good knowledge regarding informed consent [2]. Another similar study in Sudan revealed that 46% of clients had good knowledge of informed consent [15, 16]. In Rwanda, only 5% of patients had a high level of knowledge, 12% had moderate, and the rest 83% of the patients had a low level of knowledge towards informed consent [17]. A study done in Kenya revealed that knowledge regarding informed consent is limited, 46% of the patients stated that the purpose of informed consent is for hospital protection and 41% of them stated their wishes [17].

In Ethiopia, the magnitude of good knowledge of informed consent among surgical patients is low ranging from 10.5% [13] to 46.9% [18].

Client perception towards informed consent includes perception of the importance and function of consent forms, the legal and ethical status of consent, and the scope of consent [18–21]. Research in the different countries indicated that the perception of clients towards informed consent is low. A study done in Saudi Arabia indicated that 23.7% of the clients had poor perceptions of informed consent [18, 22]. In Rwanda, 23% of patients experience poor perception, and 50% and 31% of clients had moderate and high levels of perception towards surgical informed consent [17, 18].

The magnitude of client perception towards informed consent in Ethiopia among post-operated patients is low, ranging from 13.7 to 66.8% [16, 18].

Factors affecting knowledge and perception of patient informed consent in surgical procedures are level of education, residence, age, history of signing before, type of surgery, marital status, and occupation significant variables [2, 13, 17].

Many patients around the world, particularly in developing countries undergo surgery without the knowledge of the reason for the surgery, the type of surgery, and identifying the identity of the surgeon [13, 23]. The consequence of poor knowledge and perception of clients towards informed consent is patient dissatisfaction, feeling low power in their determination, low control, patient anxiety, and unaccountable for the management [18, 20, 21, 24].

Despite patient knowledge and perception of informed consent being one of the priority concerns in surgical procedures, the problem still exists in Ethiopia. In addition, studies in small-scale findings are inconsistent and inconclusive about the knowledge, perception, and determinants of informed consent. Therefore, the purpose of this systematic review and meta-analysis study was to determine the pooled prevalence and factors of knowledge and perception of patients towards informed consent among surgical patients in Ethiopia. The findings of this nationwide study will generate evidence with implications to improve physician intervention, health facility managers, and policymakers to establish guidelines for informed consent practice.

## Methods

### Study design and protocol registration

A Systematic Review and Meta-Analysis (SRMA) was conducted to quantify the pooled level of patient knowledge and perception towards informed consent and determinants among surgical patients in Ethiopia. A preliminary assessment was done to check whether a similar study was performed or not through Prospero, Epistemonikos, Semantic Scholar, and PubMed and there was no similar study. We prepared this systematic review and meta-analysis according to the preferred Reporting Items for Systematic Review and Meta-Analysis (PRISMA-2020) follow diagrams (S1 Table 1). The protocol was registered at Prospero with number CRD42023445409 and is available from: https://www.crd.york.ac.uk/PROSPERO/#myprospero.

**Table 1:** Characteristics of studies included in this systemic review and meta-analysis, in Ethiopia, 2023 (n = 7)

Author	Study period	Region	Study design	Sample size	Prevalence	Quality score
Lemmu et al. [13]	2018	Addis Ababa	Cross-sectional	385	10.5	7
Daniel et al. [16]	2022	Southern Ethiopia	Cross-sectional	423	44.4	8
Gebrehiwot et al. [18]	2021	Amhara	Cross-sectional	422	46.9	7
Nurhusien Nuru Yesuf et al. [20]	2018	Amhara	Cross-sectional	302	36	7
Kebede et al. [26]	2020	Oromia	Cross-sectional	372	22.8	6
Biyazin et al. [25]	2020	Oromia	Cross-sectional	372	22.8	7
Tsegahun Amilaku et al. [28]	2022	Southern Ethiopia	Cross-sectional	414	40.6	9

### Search strategies

We searched major databases such as PubMed, Hinary, MEDLINE, Cochrane Library, EMBASE, Scopus, African Journal Online (AJO), Semantic Scholar, Google Scholar, google, and reference lists. Besides this, University databases in the country were also searched from August 20, 2023, until September 30, 2023,. Studies conducted between January 01, 2015 to September 30, 2023, were included.

This systematic review and meta-analysis used PECO (Population, Exposure, Comparison, and outcome) to identify eligible studies. The study population (P) are surgical patients, exposure (E) associated factors, comparison (C) reference of the factors, and outcome (O) level of knowledge and perception towards informed consent. Boolean operators “OR” and “AND” were used to combine search terms. Keywords used to search includes knowledge, perception, patient, client, “informed consent”, consent, factors, determinants, predictors, “surgical patient”, “post operated patient”, “after surgery”, and Ethiopia.

Studies obtained by the reviewers’ search strategy were exported into EndNote for management. All duplicated studies obtained for different database searches were excluded. Studies eligibility was assessed first from the title, then the abstract, and finally, a full-text review was performed.

### Eligibility criteria

All observational studies (cross-sectional, case-control, and cohort) on patient knowledge and perception towards informed consent among surgical patients conducted in Ethiopia were included. Both published and unpublished studies reported the prevalence of patient knowledge and perception toward informed consent and its associated factors were included. All studies reported in English were included. Studies conducted between January 01, 2015 to September 30, 2023 were included. Articles that cannot access full text after failing to contact the primary authors were excluded.

### Outcome measurement

This systematic review and meta-analysis measured three main outcomes. The first outcome of the study was to estimate the pooled level of appropriate knowledge towards informed consent. The second outcome was to estimate the pooled level of perception towards informed consent. The third outcome was the associated factors with knowledge of informed consent among surgical patients. The level of knowledge towards informed consent was measured by 12 items and the level of perception by 8 items of questions. Patients who scored less than the mean for knowledge and perception questions had poor knowledge and poor perception respectively.

### Data extraction

The selection of studies in all the searched databases was conducted by three authors (YT, NK, and FDB) independently. The primary author, study year, year of publication, regions where the study was done, study design, sample size, prevalence, response rate, method of outcome measurement, all associated factors odds ratio, relative risk, lower confidence interval, and upper confidence interval were extracted by using Microsoft Excel format. The corresponding author was supportive of clarification on the inclusion criteria. Disagreements among data extractors were solved by consensus.

### Quality assessment and risk of bias

Three reviewers (MMM, NK, and YT) independently screened the articles that fulfilled the inclusion criteria to avoid the risk of bias. The Newcastle-Ottawa Scale (NOS) checklist was used to appraise the quality of the studies. The tool includes three parts. The first part included methodology [5] rate with five stars, the second part was comparability [2] rate with two stars and the third part was outcome with statistical test [3] rate with three stars (S2 Table). Three authors (MMM, NK, and YT) independently assessed the quality of the studies. Disagreements among reviewers were resolved by consensus and a third party (FDB).

### Data processing and analysis

Data were extracted by using Microsoft Excel format and imported into STATA version 17 for processing and analysis. The pooled prevalence of patient knowledge and perception towards informed consent was estimated by random effect model meta-analysis. The heterogeneity of the studies was assessed by observing the p-value and I^2^ statistics test. Factors associated with patient knowledge for informed consent were estimated by a log odds ratio at 95% CI. The potential source of heterogeneity was identified by subgroup analysis. In addition, Egger’s test statics and funnel plot were performed to identify potential publication bias among the included studies. The result of this meta-analysis was presented by tables, funnel plots, forest plots, and narrations.

## Results

**Fig. 1:**
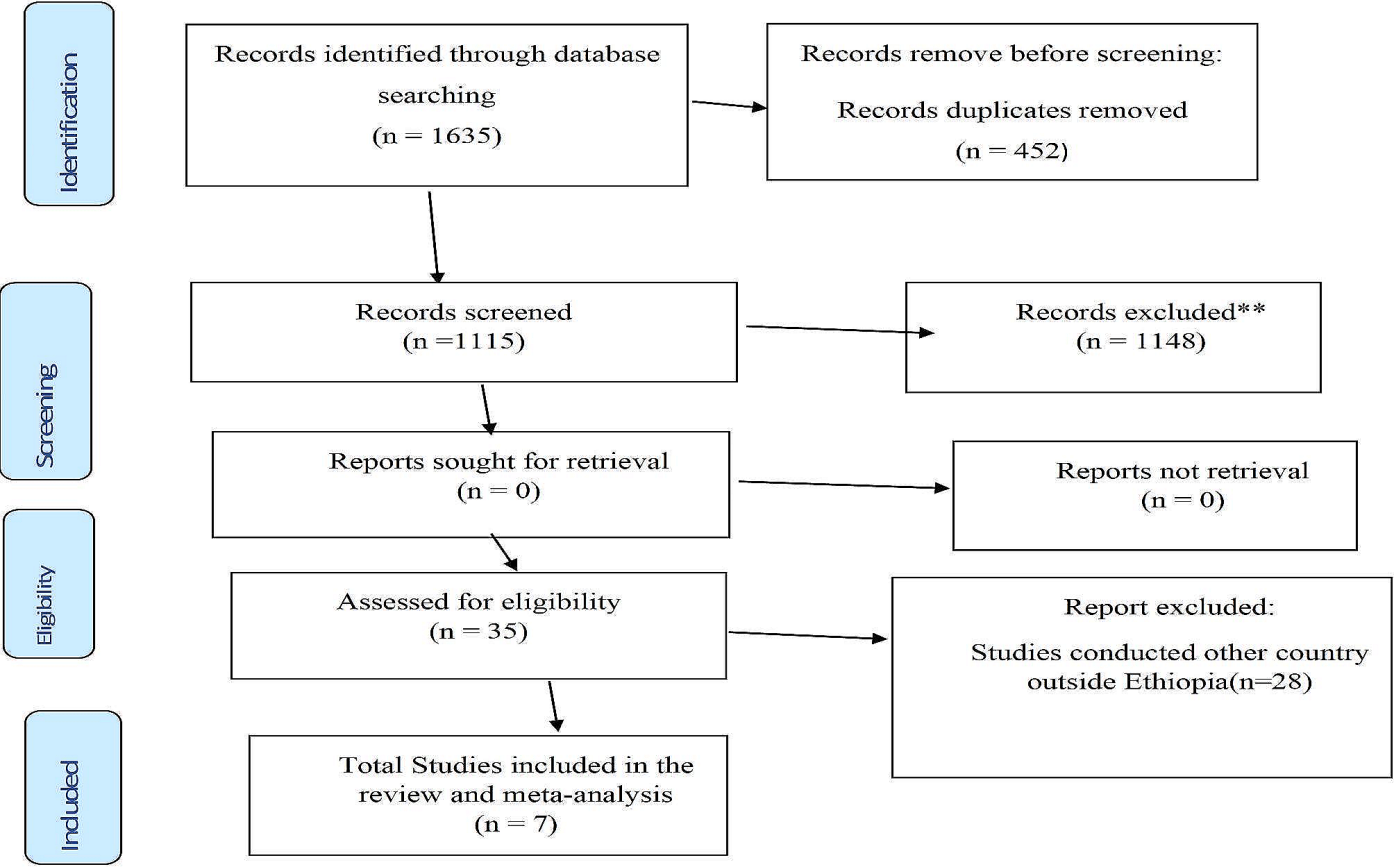
PRISMA 2020 flow diagram

A total of 1635 studies were searched by using a searching strategy in this systematic review and meta-analysis study. Among those studies, 452 articles were excluded due to redundancy. From the remaining 1115 articles, 1148 studies were excluded in the review of the abstract and title of the study that did not report the level of patient knowledge or perception and its determinants. Of the studies, 28 articles were excluded because of the study location outside Ethiopia. Finally, seven studies were included in this systematic review and meta-analysis study that met the minimum eligibility criteria (Fig. 1). Of those articles, seven studies estimated pooled level of knowledge [13, 15, 18, 20, 25–27], and four studies estimated the prevalence of perception [16, 18, 20, 28].

### Characteristics of the included studies characteristics of the included studies

From all included seven articles 2,690 study participants were used to estimate the pooled level of patient knowledge of informed consent among surgical patients in Ethiopia. The maximum sample size was 423 [16] and the minimum sample size was 302 [11]. All included studies are cross-sectional study design. The prevalence of patient knowledge of informed consent ranges from 10.5% [13] to 46.9% [18] (Table 1).

### Prevalence of patient knowledge and perception for informed consent among surgical patent in Ethiopia

**Fig. 2:**
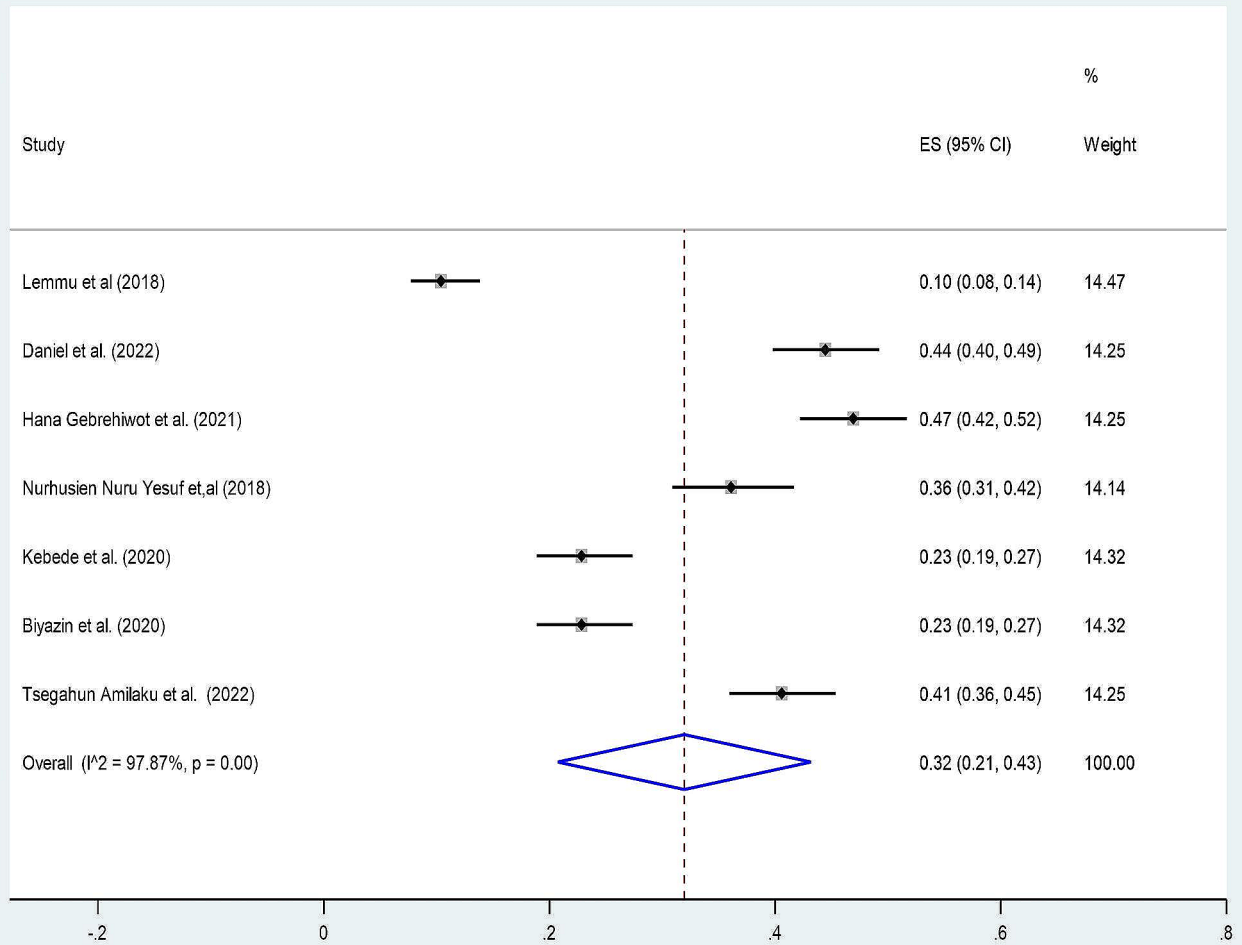
Forest plot pooled prevalence of patient knowledge towards informed consent among surgical patients in Ethiopia 2023

**Fig. 3:**
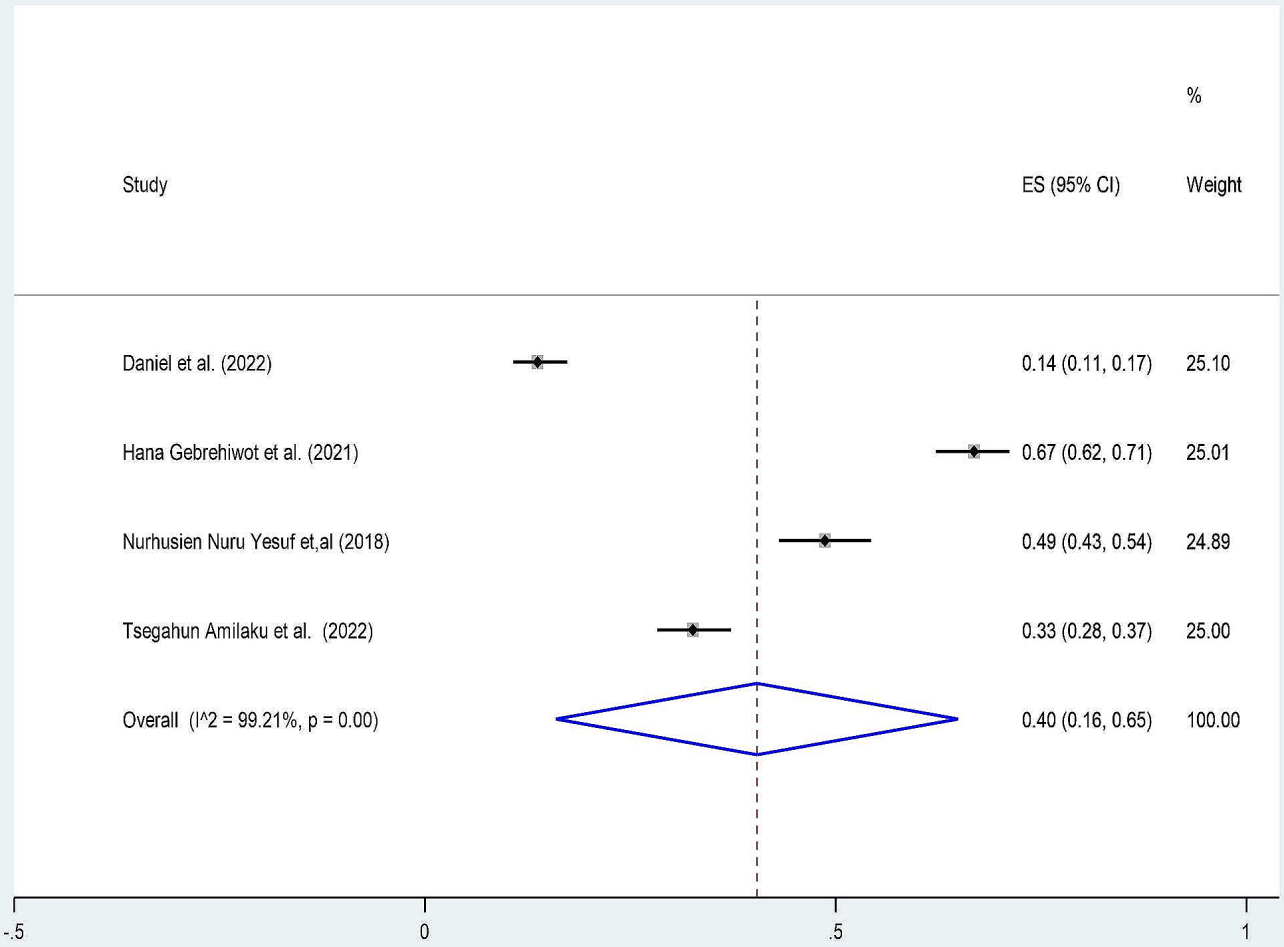
Forest plot pooled prevalence of patient perception towards informed consent among surgical patients in Ethiopia 2023

We observed that there is a variation in the prevalence of patient knowledge and perception of informed consent among surgical patients in Ethiopia. A random effect meta-analysis model for seven studies pooled prevalence of patient knowledge for informed consent was 32% (95% CI: 21, 43) with (I^2^ = 97.87% and p_value < 0.001) (Fig. 2). Similarly, four studies pooled the prevalence of perception of patients towards informed consent at 40% (95% CI: 16, 65) with (I^2^ = 99.21% and p_value < 0.001) (Fig. 3).

**Fig. 4:**
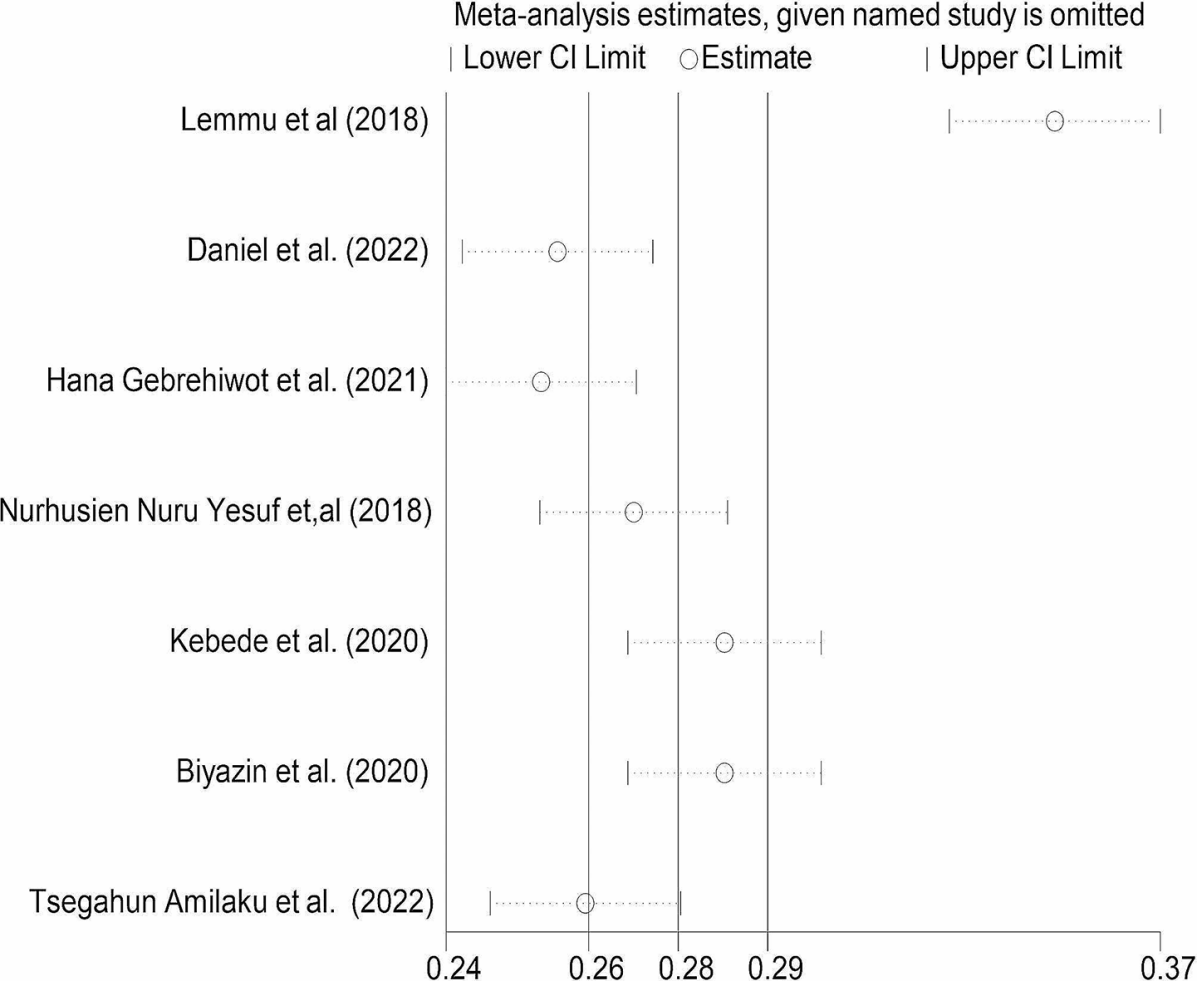
Forest plot for patient knowledge towards informed consent among surgical patients in Ethiopia, sensitivity analysis, 2023

To identify potential causes of publication bias among the included studies Egger’s test statistics and funnel plot were performed. As a result, the funnel plot indicated that there was asymmetric distribution in the included studies. In addition, Egger’s test statics indicated that there was evidence to show publication bias (p = 0.009) with a standard error of 7.39. Besides this, we performed a sensitivity analysis to identify any outlier that causes a source of heterogeneity to estimate the pooled prevalence of patient knowledge of informed consent among surgical patients in Ethiopia. The finding indicated that there was one outlier study far apart from the confidence interval of the rest included studies. As a result, we were confident enough, that in this systematic review and meta-analysis study, there was a single study that affected the overall pooled prevalence of patient knowledge of informed consent among surgical patients in Ethiopia (Fig. 4).

**Fig. 5:**
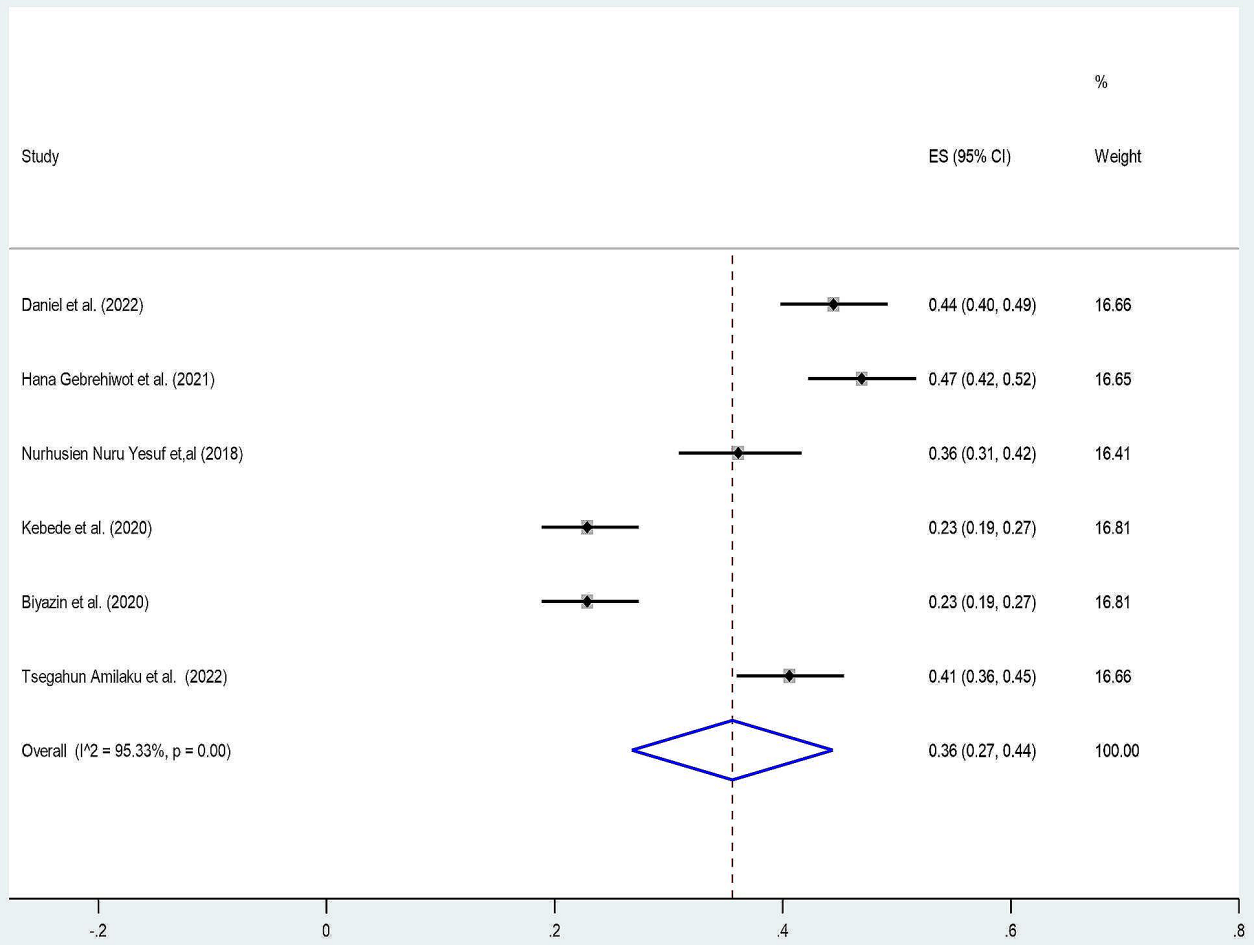
Forest plot pooled prevalence of patient knowledge towards informed consent among surgical patients after omitting a single outlier study in Ethiopia 2023

Accordingly, we omitted a single study that lies outside the confidence interval and performed a random effect meta-analysis model in six studies. The pooled level of patient knowledge of informed consent after removing one study changed from 32 to 36% (95% CI: 27,44) with (I^2^ = 95.33% and p < 0.00) (Fig. 5). In addition, the funnel plot changes are somehow symmetrical, and Egger’s test statistics result also revealed that there was no evidence of publication bias (p = 0.17) with a standard error of 20.56.

### Subgroup analysis

Subgroup analysis was performed by using sample size, study period, and region of the study to identify the potential source of heterogeneity. As a result, studies conducted after 2020 were the possible cause of heterogeneity with the higher pooled prevalence estimated which was 44% (95% CI: 40, 48). Besides this, studies conducted in the Oromia region were other sources of heterogeneity with a lower pooled prevalence of 23% (95% CI: 20,26) (Table 2).

**Table 2:** Subgroup analysis of the pooled prevalence of patient knowledge towards informed consent in Ethiopia, 2023 (n = 7)

Variables	Subgroup	Number of studies	Prevalence of subgroup with 95% CI	I^2^ (%)	P
Region	Southern Ethiopia	2	43% (39%, 46%)	–	–
	Amhara	2	42% (39%, 46%)	–	–
	Oromia	2	23% (20%, 26%)	–	–
	Others	1	10% (8%, 14%)		
Year of the study	≤ 2020	4	23% (13%, 33%)	96%	< 0.001
	> 2020	3	44% (40%, 48%)	–	–
Sample size	≥ 385	4	36% (16%, 55%)	98.85%	< 0.001
	< 385	3	27% (19%, 35%)	–	–

### Factors affected patient knowledge of informed consent among surgical patients in Ethiopia

The factors of residence, formal education, history of signed informed consent before, and type of surgery were investigated in the pooled effect on patient knowledge of informed consent.

The association between formal education and patient knowledge towards informed consent was examined by using three studies, of which there was no association [26] and the rest two were positive associations with patient knowledge towards informed consent [13, 16]. Hence, there was a positive relationship between formal education and patient knowledge of informed consent. The pooled effect of appropriate patient knowledge of informed consent is nearly three times more likely among formally educated patients than counterparts 2.69 (95% CI: 1.18, 6.15) (Table 3).

**Table 3:** Effect of formal education, history of informed consent signed before, residence, and type of surgery on patient knowledge towards informed consent in Ethiopia; systematic review and meta-analysis

Study		Estimated effect	95% CI	Weight
Formal education	Lemmu et al. (2018)	4.80	0.05, 483.78	3.20
	Daniel et al. (2022)	3.26	1.30, 8.17	80.80
	Kebede et al. (2020)	0.92	0.12, 7.22	16.00
	Pooled effect	**2.69**	**1.18, 6.15**	100
Residence	Lemmu et al. (2018)	4.70	0.12, 187.93	12.43
	Daniel et al. (2022)	0.25	0.00, 12.81	10.82
	Nurhusien Nuru Yesuf et al. (2018)	1.52	0.18, 12.62	37.76
	Kebede et al. (2020)	0.69	0.09, 5.54	38.99
	Pooled effect	1.06	0.29, 3.87	100
Informed consent signed before	Daniel et al. (2020)	4.06	0.70, 23.52	52.26
	Nurhusien Nuru Yesuf et al. (2018)	2.20	0.12, 41,57	18.92
	Tsegahun Amilaku et al. (2022)	4.21	0.36, 45.87	28.13
	Pooled effect	**3.65**	**1.02, 13.11**	100
Type of surgery	Lemmu et al. (2018)	0.96	0.07, 13.08	39.54
	Kebede et al. (2020)	0.73	0.09, 6.08	60.46
	Pooled effect	0.81	0.16, 4.21	100

Similarly, we examined the association between having history of signed informed consent before and patient knowledge of informed consent by using three studies [16, 20, 27]. Accordingly, there was a statistically positive relation between history of sign before and patient knowledge of informed consent. Patients who had experienced signing informed consent before the pooled effect of appropriate patient knowledge were more than three times more likely than had no history of signing before 3.65 (95% CI:1.02,13.11) (Table 3).

In this meta-analysis, the pooled effect of residence on patient knowledge of informed consent was examined by using four studies. Of which being urban residence, 2 studies had no effect [16, 26] and 2 had a positive relation with patient knowledge of informed consent [13, 20]. As a result, there was no statistically significant pooled effect of residence on patient knowledge of informed consent 1.06 (95% CI: 0.26, 3.87) (Table 3).

Finally, the pooled effect of the type of surgery on patient knowledge of informed consent was assessed using two studies [13, 26]. The result of these two studies indicated that there was no statistically significant pooled effect of type of surgery on patient knowledge of informed consent among surgical patients 0.81(95% CI:0.16,4.21) (Table 3).

## Discussion

Patient knowledge and perception of informed consent are important to increase client satisfaction and better health outcomes for surgical patients. Evidence on patient knowledge, perception, and its determinants is crucial for physicians, health managers, and policymakers. Therefore, this systematic review and meta-analysis were performed by using available primary studies in Ethiopia. The finding of the study revealed that the pooled prevalence of appropriate patient perception of informed consent was 40% (95% CI: 16%, 65%) among surgical patients in Ethiopia. This finding was congruent with studies conducted in Egypt 27.3% [3] and in South Africa 27% of patients perceived signed consent with understanding [29]. However, the result of this finding was lower than the study conducted in Nigeria 97% of patients were satisfied with the explanation of informed consent [30] and University of Colorado on repeat back and no repeat back participants, favorable perception of patients towards informed consent was 88% [31]. The possible justification for this variation might be due to the different methods of the study, the sample size in Nigeria was 398 whereas this study incorporates 2690 participants in the primary study.

The pooled prevalence of appropriate patient knowledge of informed consent was 32% (95% CI:21, 43) among surgical patients in Ethiopia. This finding was incongruent with the study finding in German 32.6% of patients correctly answered knowledge questions [32]. However, this finding is higher than the study done in Rwanda 5% of the participants had a high level of knowledge, 12% moderate, and the rest 83% had a low level of knowledge towards informed consent [17]. The possible reason for this discrepancy might be due to the difference in sample size in Rwanda was 147 and it was conducted in one military hospital. However, this finding was lower than a systematic review study done in Pakistan 50% [33], India 68% understood the type and consequence of the study [34], Portuguese 44.7%, Croatia level of knowledge average, and 60% had partial knowledge [35]. These variations might be due to the difference in the educational status of study participants, differences in economic status, and giving value for informed consent during surgical producer of the patient. It may vary the culture and behavior of physicians who focus on informed consent. Developed countries have a high-level concern for patient rights and informed consent; whereas in developing countries including Ethiopia focus on patient rights is limited.

Subgroup analysis was performed by taking the study setting, sample size, and study period. In this regrade, the study conducted after 2020 indicated a source of heterogeneity of 44% (95% CI: 40, 48) as compared to studies conducted before or in 2020. This implies that as the period of study increases the patient knowledge towards informed consent also increases. This variation might be explained as the period of study is more recent the patient may get more information about informed consent. It might be due to the increase in the number of health professionals from time to time who had room to explain informed consent. In addition, studies conducted with a sample size greater than or equal to 385 were another source of heterogeneity of 36% (95% CI: 16, 55) than a sample size less than 385. This difference might be as the sample size increases and also increases the representativeness of the finding.

The pooled effects of patient knowledge towards informed consent among formally educated patients were 2.69 times more likely than counterparts (Table 3). This finding is in line with the study in South Africa [29], Pakistan [36], and India [24]. The possible explanation for this finding might be those educated patients can easily understand the physician’s explanation of informed consent [37]. There may be a language barrier to the understanding of the consent formats.

For patients who had experienced signed informed consent before, the pooled effect of patient knowledge towards informed consent was 3.65 times more likely than those not signed before. This finding is consistent with a systematic review done on client comprehension; those patients demonstrated the highest understanding of informed consent (Systematic review) [38]. The implication of this finding is once the patient was exposed for signed informed consent, had more understanding. Besides this, those patients had more knowledge of diagnosis, treatment, and possible outcomes of treatment.

This meta-analysis revealed that there is no statistically significant pooled effect residence on patient knowledge towards informed consent in Ethiopia. In addition, the type of surgery had no statistically significant pooled effect on patient knowledge of informed consent.

The limitation of this study primary studies included in this meta-analysis were found to be in Southern Ethiopia, Amhara, Oromia, and Addis Ababa city, which is under-represented in other regions in the country. In addition, a limited number of primary studies are available in Ethiopia. Besides this, only a few systematic review and meta-analysis studies on patient knowledge and perception of informed consent to compare the findings.

## Conclusion

The appropriate patient knowledge and perception of informed consent in Ethiopia is low. Formal education and history of signed informed consent were positive factors for the level of patient knowledge of informed consent in Ethiopia. Physicians, policymakers, and health facility managers should focus on patients without prior experience with signed informed consent and not had formal education to improve patient knowledge towards informed consent. Physicians should provide clear information regarding the content of informed consent those patients had no formal education and experience before to increase their knowledge of informed consent.

### Electronic supplementary material

Below is the link to the electronic supplementary material.

Supplementary Material 1

Supplementary Material 2

## Data Availability

The datasets used and analyzed during the current study are available from the first author.
