# Supplementary material for: Patient knowledge of surgical informed consent and shared decision-making process among surgical patients in Ethiopia: a systematic review and meta-analysis
Source: Patient Saf Surg. 2024 Jan 13;18:2. doi: 10.1186/s13037-023-00386-5 (PMC10787976; doi:10.1186/s13037-023-00386-5)
Supplement: Supplementary file 1 — Supplementary Material 1 [file 13037_2023_386_MOESM1_ESM.docx]

| **Section and Topic** | **Item #** | **Checklist item** | **Location where item**  **is reported** |
| --- | --- | --- | --- |
| **TITLE:** **Determinants of patient knowledge and perception towards informed consent among surgical patients in Ethiopia: Systematic Review and Meta-Analysis** | | |  |
| Title | 1 | Identify the report as a systematic review. |  |
| **Background:** Informed consent is the safeguarding of the patient in medical practice at different standards such as ethical, legal, and administrative purposes. Patient knowledge and perception of informed consent are one of the priority concerns in surgical procedures. Patient knowledge and perception towards informed consent increased patient satisfaction, feeling high power on their determination, accountability for the management, and facilitating positive treatment outcomes. Despite this, in Ethiopia, there are small-scale primary studies with inconsistent and inconclusive findings. Therefore, this systematic review and meta-analysis study estimated the pooled prevalence of patient knowledge and perception of informed consent and its determinants in Ethiopia.  **Methods**: We searched major databases such as PubMed, Hinary, MEDLINE, Cochrane Library, EMBASE, Scopus, African Journal Online (AJO), Semantic Scholar, Google Scholar, google, and reference lists. Besides this, University databases in the country were also searched from August 20/2023/ to September 30/2023. All published and unpublished studies that report the prevalence of patient knowledge and perception toward informed consent and its associated factors were included. There are three outcome measurements pooled level of patient knowledge towards informed consent, pooled level of patient perception towards informed consent, and pooled effect that affects patient knowledge of informed consent. Three reviewers (MMM, NK, and YT) independently screened the articles that fulfilled the inclusion criteria to avoid the risk of bias. The quality of the studies was appraised by using a modified version of the Newcastle-Ottawa Scale (NOS).  **Results:** The pooled prevalence of good patient knowledge and perception towards informed consent was 32% (95% CI: 21, 43) and 40% (95% CI: 16, 65) respectively. Having formal education 2.69 (95% CI: 1.18, 6.15) and history of signed informed consent before 3.65 (95% CI:1.02,13.11) had a statistically significant association with good patient knowledge towards informed consent.  **Conclusion:** The level of good patient knowledge and perception of informed consent in Ethiopia is low. Formal education and history of signed informed consent were positive factors affecting the level of patient knowledge of informed consent in Ethiopia. Physicians, policymakers, and health facility managers should focus on those patients who have no experience signing informed consent and do not have formal education to improve patient knowledge towards informed consent. | | |  |
| Abstract | 2 | See the PRISMA 2020 for Abstracts checklist. |  |
| **INTRODUCTION**: Informed consent is the process of decision-making made by the client or his/her surrogates after fully understanding of what he/she is consenting (1). It is a voluntary agreement by a competent individual after adequate information regarding the procedure performed, potential benefits and risks, and alternative options of management to make decisions without corrosion (2). One of the medical practices associated with high risks that require informed consent is surgical invasive procedures (3). The patient has the right to obtain appropriate expression of all risks and benefits, type of producer, options of treatment, and consequences with scientific justification and evidence (4). One of the fundamental pillars of surgical treatment is the informed consent of the patient (5). A globally recognized safeguard for clients undergoing invasive procedures is informed consent (6).  Many patients around the world, particularly in developing countries undergo surgery without the knowledge of the reason for the surgery, the type of surgery, and identifying the identity of the surgeon (11).  Despite patient knowledge and perception of informed consent being one of the priority concerns in surgical procedures, the problem still exists in Ethiopia. In addition, studies in small-scale findings are inconsistent and inconclusive about the knowledge, perception, and determinants of informed consent. Therefore, the purpose of this systematic review and meta-analysis study was to determine the pooled prevalence and factors of knowledge and perception of patients towards informed consent among surgical patients in Ethiopia. The finding of this nationwide study will generate evidence with implications to improve physician intervention, health facility managers, and policymakers to establish guidelines for informed consent practice | | |  |
| Rationale | 3 | Describe the rationale for the review in the context of existing knowledge. |  |
| Objectives | 4 | Provide an explicit statement of the objective(s) or question(s) the review addresses. |  |
| METHODS: A Systematic Review and Meta-Analysis (SRMA) was conducted to quantify the pooled level of patient knowledge and perception towards informed consent and determinants among surgical patients in Ethiopia. A preliminary assessment was done to check whether a similar study was performed or not through Prospero, Epistemonikos, Semantic Scholar, and PubMed and there was no similar study. We prepared this systematic review and meta-analysis according to the preferred Reporting Items for Systematic Review and Meta-Analysis (PRISMA-2020) following diagrams.  Inclusion criteria: All observational studies (cross-sectional, case-control, and cohort) both published and unpublished reports on the prevalence of patient knowledge and perception towards informed consent and its associated factors among surgical patients conducted in Ethiopia were included. All studies reported in English were included.  Exclusion Criteria: Studies that did not measure the outcome variable and that could not access full text after failing to contact the primary authors were excluded.  We searched major databases such as PubMed, Hinary, MEDLINE, Cochrane Library, EMBASE, Scopus, African Journal Online (AJO), Semantic Scholar, Google Scholar, google, and reference lists. Besides this, University databases in the country were also searched from August 20/2023/ to September 30/2023. Keywords used to search includes knowledge, perception, patient, client, “informed consent”, consent, factors, determinants, predictors, “surgical patient”, “post operated patient”, “after surgery”, and Ethiopia.  **Outcome measurement:** This systematic review and meta-analysis measured three main outcomes. The first outcome of the study was to estimate the pooled level of good knowledge towards informed consent. The second outcome was to estimate the pooled level of perception towards informed consent. The third outcome was the associated factors with knowledge of informed consent among surgical patients.  The quality of the studies was appraised by using a modified version of the Newcastle-Ottawa Scale (NOS). Three authors (MMM, NK, and YT) assessed the quality of the study independently. Disagreements among reviewers were resolved by consensus and a third party (FDB).  The selection of studies in all the searched databases was conducted by three authors (YT, NK, and FDB) independently. The primary author, study year, year of publication, regions where the study was done, study design, sample size, prevalence, response rate, method of outcome measurement, all associated factors odds ratio, relative risk, lower confidence interval, and upper confidence interval were extracted by using Microsoft Excel format.  Extracted data was imported into STATA version 17 for processing and analysis. The pooled level of knowledge and perception towards informed consent was estimated by random effect model meta-analysis. The heterogeneity of the studies was assessed by observing the p-value and I^2^ statistics test. Factors associated with patient knowledge for informed consent were estimated by a log odds ratio at 95% CI. To identify possible sources of heterogeneity subgroup analysis was performed. In addition, the funnel plot and Egger’s test were conducted to identify potential publication bias in the included studies. The result of this meta-analysis was presented by tables, funnel plots, forest plots, and narrations. | | |  |
| Eligibility criteria | 5 | Specify the inclusion and exclusion criteria for the review and how studies were grouped for the syntheses. |  |
| Information sources | 6 | Specify all databases, registers, websites, organizations, reference lists, and other sources searched or consulted to identify studies. Specify the date when each source was last searched or consulted. |  |
| Search strategy | 7 | Present the full search strategies for all databases, registers, and websites, including any filters and limits used. |  |
| Selection process | 8 | Specify the methods used to decide whether a study met the inclusion criteria of the review, including how many reviewers screened each record and each report retrieved, whether they worked independently, and if applicable, details of automation tools used in the process. |  |
| Data collection process | 9 | Specify the methods used to collect data from reports, including how many reviewers collected data from each report, whether they worked independently, any processes for obtaining or confirming data from study investigators, and if applicable, details of automation tools used in the process. |  |
| Data items | 10a | List and define all outcomes for which data were sought. Specify whether all results that were compatible with each outcome domain in each study were sought (e.g. for all measures, time points, analyses), and if not, the methods used to decide which results to collect. |  |
|  | 10b | List and define all other variables for which data were sought (e.g. participant and intervention characteristics, funding sources). Describe any assumptions made about any missing or unclear information. |  |
| Study risk of bias assessment | 11 | Specify the methods used to assess the risk of bias in the included studies, including details of the tool(s) used, how many reviewers assessed each study whether they worked independently, and if applicable, details of automation tools used in the process. |  |
| Effect measures | 12 | Specify for each outcome the effect measure(s) (e.g. risk ratio, mean difference) used in the synthesis or presentation of results. |  |
| Synthesis methods | 13a | Describe the processes used to decide which studies were eligible for each synthesis (e.g. tabulating the study intervention characteristics and comparing against the planned groups for each synthesis (item #5)). |  |
|  | 13b | Describe any methods required to prepare the data for presentation or synthesis, such as handling of missing summary statistics, or data conversions. |  |
|  | 13c | Describe any methods used to tabulate or visually display the results of individual studies and syntheses. |  |
|  | 13d | Describe any methods used to synthesize results and provide a rationale for the choice(s). If meta-analysis was performed, describe the model(s), method(s) to identify the presence and extent of statistical heterogeneity, and software package(s) used. |  |
|  | 13e | Describe any methods used to explore possible causes of heterogeneity among study results (e.g. subgroup analysis, meta-regression). |  |
|  | 13f | Describe any sensitivity analyses conducted to assess the robustness of the synthesized results. |  |
| Reporting bias assessment | 14 | Describe any methods used to assess the risk of bias due to missing results in a synthesis (arising from reporting biases). |  |
| Certainty assessment | 15 | Describe any methods used to assess certainty (or confidence) in the body of evidence for an outcome. |  |

| **Section and Topic** | **Item #** | **Checklist item** | **Location where item**  **is reported** |
| --- | --- | --- | --- |
| RESULTS: From all included seven articles 2,690 study participants were used to estimate the pooled level of patient knowledge of informed consent among surgical patients in Ethiopia. The maximum sample size was 423 (16) and the minimum sample size was 302 (11). All included studies are cross-sectional study design. The prevalence of patient knowledge of informed consent ranges from 10.5% (13) to 46.9% (18) (**Table 1**).  A random effect meta-analysis model for seven studies pooled prevalence of patient knowledge for informed consent was 32% (95% CI: 21, 43) with (I^2^=97.87% and p_value <0.001) (**Figure 2**). Similarly, four studies pooled the prevalence of perception of patients towards informed consent 40% (95% CI: 16, 65) with (I^2^=99.21% and p_value <0.001) (**Figure 3**).  To identify potential causes of publication bias among the included studies Egger’s test statistics and funnel plot were performed. As a result, the funnel plot indicated that there was asymmetric distribution in the included studies. In addition, Egger’s test statics indicated that there was evidence to show publication bias (p=0.009) with a standard error of 7.39. Subgroup analysis was performed by using sample size, study period, and region of the study to identify the potential source of heterogeneity. As a result, studies conducted after 2020 were the possible cause of heterogeneity with the higher pooled prevalence estimated 44% (95% CI: 40, 48). Besides this, studies conducted in the Oromia region were other sources of heterogeneity with a lower pooled prevalence of 23% (95% CI: 20,26) (**Table 2**).  Formal education and signed informed consent had a statistically significant pooled effect on patient knowledge of informed consent among surgical patients in Ethiopia. | | |  |
| Study selection | 16a | Describe the results of the search and selection process, from the number of records identified in the search to the number of studies included in the review, ideally using a flow diagram. |  |
|  | 16b | Cite studies that might appear to meet the inclusion criteria, but which were excluded, and explain why they were excluded. |  |
| Study characteristics | 17 | Cite each included study and present its characteristics. |  |
| Risk of bias in studies | 18 | Present assessments of risk of bias for each included study. |  |
| Results of individual studies | 19 | For all outcomes, present, for each study: (a) summary statistics for each group (where appropriate) and (b) an effect estimate and its precision (e.g. confidence/credible interval), ideally using structured tables or plots. |  |
| Results of syntheses | 20a | For each synthesis, briefly summarise the characteristics and risk of bias among contributing studies. |  |
|  | 20b | Present results of all statistical syntheses conducted. If meta-analysis was done, present for each the summary estimate and its precision (e.g. confidence/credible interval) and measures of statistical heterogeneity. If comparing groups, describe the direction of the effect. |  |
|  | 20c | Present results of all investigations of possible causes of heterogeneity among study results. |  |
|  | 20d | Present results of all sensitivity analyses conducted to assess the robustness of the synthesized results. |  |
| Reporting biases | 21 | Present assessments of risk of bias due to missing results (arising from reporting biases) for each synthesis assessed. |  |
| Certainty of evidence | 22 | Present assessments of certainty (or confidence) in the body of evidence for each outcome assessed. |  |
| **DISCUSSION:** The finding of the study revealed that the pooled prevalence of good patient perception of informed consent was 40% (95% CI: 16%, 65%) among surgical patients in Ethiopia. This finding was congruent with studies conducted in Egypt 27.3% (3) and in South Africa 27% of patients perceived signed consent with understanding (29). However, the result of this finding was lower than the study conducted in Nigeria 97% of patients were satisfied with the explanation of informed consent (30) and University of Colorado on repeat back and no repeat back participants, favorable perception of patients towards informed consent was 88% (31). The possible justification for this variation might be due to the different methods of the study, the sample size in Nigeria was 398 whereas this study incorporates 2690 participants in the primary study.  The pooled prevalence of good patient knowledge of informed consent was 32% (95% CI:21, 43) among surgical patients in Ethiopia. This finding was incongruent with the study finding in German 32.6% of patients correctly answered knowledge questions (32). However, this finding is higher than the study done in Rwanda 5% of the participants had a high level of knowledge, 12% moderate, and the rest 83% had a low level of knowledge towards informed consent (17). The possible reason for this discrepancy might be due to the difference in sample size in Rwanda was 147 and it was conducted in one military hospital. However, this finding was lower than a systematic review study done in Pakistan 50% (33), India 68% understood the type and consequence of the study (34), Portuguese 44.7%, Croatia level of knowledge average, and 60% had partial knowledge (35). These variations might be due to the difference in the educational status of study participants, differences in economic status, and giving value for informed consent during surgical producer of the patient. It may vary the culture and behavior of physicians who focus on informed consent. Developed countries have a high-level concern for patient rights and informed consent; whereas in developing countries including Ethiopia focus on patient rights is limited.  The pooled effects of patient knowledge towards informed consent among formally educated patients were 2.69 times more likely than counterparts (**Table 3**). This finding is in line with the study in South Africa (29), Pakistan (36), and India (24). The possible explanation for this finding might be those educated patients can easily understand the physician's explanation of informed consent. There may be a language barrier to the understanding of the consent formats.  For patients who had experienced signed informed consent before, the pooled effect of patient knowledge towards informed consent was 3.65 times more likely than those not signed before. This finding is consistent with a systematic review done on client comprehension; those patients demonstrated the highest understanding of informed consent (Systematic review). The implication of this finding is once the patient was exposed for signed informed consent, had more understanding. Besides this, those patients had more knowledge of diagnosis, treatment, and possible outcomes of treatment. | | |  |
| Discussion | 23a | Provide a general interpretation of the results in the context of other evidence. |  |
|  | 23b | Discuss any limitations of the evidence included in the review. |  |
|  | 23c | Discuss any limitations of the review processes used. |  |
|  | 23d | Discuss the implications of the results for practice, policy, and future research. |  |
| **OTHER INFORMATION:** The protocol was registered at Prospero with number CRD42023445409 and is available from: https://www.crd.york.ac.uk/PROSPERO/#myprospero.  The authors declare that they have no competing interest concerning research and authorship of this article and non-financial support for the review. Availability of data, code, and other materials used are available from the first author. | | |  |
| Registration and protocol | 24a | Provide registration information for the review, including the register name and registration number, or state that the review was not registered. |  |
|  | 24b | Indicate where the review protocol can be accessed, or state that a protocol was not prepared. |  |
|  | 24c | Describe and explain any amendments to information provided at registration or in the protocol. |  |
| Support | 25 | Describe sources of financial or non-financial support for the review, and the role of the funders or sponsors in the review. |  |
| Competing interests | 26 | Declare any competing interests of review authors. |  |
| Availability of data, code, and other materials | 27 | Report which of the following are publicly available and where they can be found: template data collection forms; data extracted from included studies; data used for all analyses; analytic code; any other materials used in the review. |  |

*From:* Page MJ, McKenzie JE, Bossuyt PM, Boutron I, Hoffmann TC, Mulrow CD, et al. The PRISMA 2020 statement: an updated guideline for reporting systematic reviews. BMJ 2021;372:n71. doi: 10.1136/bmj.n71

For more information, visit: <http://www.prisma-statement.org/>
